# Triazole 187 is a biased KOR agonist that suppresses itch without sedation and induces anxiolytic-like behaviors in mice

**DOI:** 10.1038/s41386-026-02372-8

**Published:** 2026-03-09

**Authors:** Allison Volf, Tarsis F. Brust, Robin R. Kobylski, Kerri M. Czekner, Edward L. Stahl, Michael D. Cameron, Ashley E. Trojniak, Abigale B. Wood, Jeffrey Aubé, Laura M. Bohn

**Affiliations:** 1 The Skaggs Graduate School at Scripps Research, La Jolla, FL USA; 2 Department of Molecular Medicine, The Herbert Wertheim UF Scripps Institute for Biomedical Innovation & Technology, Jupiter, FL USA; 3 Department of Molecular Pharmacology and Physiology, Morsani College of Medicine, University of South Florida, Florida, FL USA; 4 Division of Chemical Biology and Medicinal Chemistry, UNC Eshelman School of Pharmacy, University of North Carolina at Chapel Hill, Chapel Hill, NC USA

**Keywords:** Pharmacology, Cell signalling

## Abstract

Kappa opioid receptor agonists are clinically used to treat pruritus and have therapeutic potential for the treatment of pain and neuropsychiatric disorders. We have previously shown that triazole 1.1 is a G protein signaling-biased KOR agonist, that can suppress itch without producing signs of sedation in mice. This profile was recapitulated in rats and non-human primates; however, triazole 1.1 had limited potency as an antipruritic. Here we describe a more potent, G protein signaling-biased agonist, triazole 187. Triazole 187 is a potent antipruritic agent and does not decrease spontaneous locomotor activity; interestingly, it produces anxiolytic-like behaviors in mice, an effect not observed for triazole 1.1. In addition to curbing sedation, triazole 187 produces only mild diuresis, resulting in 30% of urine output induced by U50,488H at a dose that is more than 100-fold the antipruritic potency dose. Compounds like triazole 187 may present a means to treat anxiety that is independent of or accompanied by persistent chronic itch while avoiding sedation and diuresis accompanied by typical KOR agonists.

## Introduction

The kappa opioid receptor (KOR) is a seven-transmembrane spanning G protein-coupled receptor (GPCR) [1] distributed throughout the central and peripheral nervous system (CNS and PNS) [2]. KOR agonists are clinically used for the treatment of non-histamine-induced itch, or pruritis [3–7], while also being pursued as a target in therapies such as pain [8, 9], anxiety [10–12], opioid abuse disorder [13–16] and as potential non-serotonergic psychedelic therapy [17, 18]. Unfortunately, activation of KOR in the CNS can lead to undesired side effects, such as diuresis, sedation, and dysphoria [19–24] which has limited the clinical utility of KOR agonists.

To avoid CNS-mediated side effects, drug development has focused on KOR agonists that are restricted to the periphery as a treatment for pruritus. These efforts have led to the FDA-approval of difelikefalin to treat pruritus associated with chronic kidney disease in adults undergoing hemodialysis [3]. While dysphoria and hallucinations have not been reported by patients treated with recommended doses of difelikefalin; drowsiness, diarrhea, and dizziness have been noted despite the “peripheral restriction” of the drug [25]. However, a recent clinical trial of difelikefalin in *notalgia paresthetica*, a neuropathic disorder characterized by pruritus in a region of the upper back, did not show meaningful clinical benefit at any dose compared to placebo and thus further clinical trials have been abandoned [26]. The KOR agonist, nalfurafine, is the only brain penetrant KOR agonist clinically available for the treatment of pruritus and, at the time of writing, is only approved in Japan and South Korea [4, 5, 27, 28]. At clinically relevant doses in humans, nalfurafine produces anti-pruritic effects without causing sedation or dysphoria [5, 29], however side effects such as drowsiness and constipation have been observed [27]. These side effects may be attributed to nonselectivity as nalfurafine is also an agonist at other receptors, such as the mu opioid receptor [5]. Interestingly, nalfurafine has revealed atypical KOR agonist pharmacology and has been described as a G protein-signaling biased agonist [5].

Biased agonism has been explored as a means to optimize therapeutic properties while avoiding certain side effects [30, 31]. We previously introduced triazole 1.1 [32] and have shown that this fully efficacious agonist preferentially promotes KOR-mediated ^35^S-GTPγS binding over βarrestin2 recruitment, adenylyl cyclase inhibition, and ERK1/2 activation relative to conventional KOR agonists U69,593 [22, 33, 34]. We have also demonstrated that the bias properties of triazole 1.1 are maintained when compared to U50,488H, a typical KOR agonist that is more suitable for in vivo evaluation [22]. Triazole 1.1 is brain penetrant and binds to KOR with high selectivity [22, 32]. In mice, triazole 1.1 is antipruritic and antinociceptive, but unlike typical KOR agonists, such as U50,488H, triazole 1.1 does not decrease spontaneous locomotor activity [22]. Such dose-dependent decreases in ambulatory behavior in mice have been shown to correlate with sedative-like drug effects in higher species [35–37] suggesting that triazole 1.1 may not be sedating. Similarly, when tested in non-human primates, triazole 1.1 does not produce sedation [16, 38]. However, higher doses of triazole 1.1 are necessary to achieve the same antipruritic effect as U50,488H in rats, indicating in vivo potency of a biased KOR agonist could be improved upon [22]. Therefore, we sought to enhance cellular potency while preserving the biased signaling profile of triazole 1.1, to determine whether in vivo potency could be improved while maintaining a broad therapeutic window between antipruritic efficacy and sedation.

In this study, we introduce triazole 187, a member of a new series of triazole agonists [39]. With the replacement of a thioether and a furan group by a carbon and a thiophenyl, respectively, triazole 187 shows improved potency in multiple signaling cascades, while retaining biased agonism for promoting ^35^S-GTPγS binding over βarrestin2 recruitment, adenylyl cyclase inhibition, and ERK1/2 activation. Like triazole 1.1, triazole 187 enters the brain when administered systemically and binds to KOR with high selectivity. Overall, triazole 187 achieves antipruritic efficacy at lower doses compared to triazole 1.1 while avoiding sedation and limiting the extent of diuresis. In addition, we find that triazole 187 produces anxiolytic-like behaviors that are not confounded by sedative properties typically seen in KOR agonists. Compounds like triazole 187 could be useful in treating pruritus as well as the negative anticipations and anxiety that accompany intractable itch. In this study, we present additional mouse behavioral evidence to support that triazole 187 may represent a new class of anxiolytics that spare sedative properties typically associated with KOR agonists.

## Materials and methods

### Chemicals and drugs

The following compounds were purchased as indicated: U69,593 from Sigma-Aldrich (St. Louis, MO); U50,488H from Tocris (Ellisville, MO); Tween 80 and dimethyl sulfoxide (DMSO) (certified grade reference material pharmaceutical secondary standard, ≥99.7% purity) from Fisher Scientific (Pittsburg, PA); and sterile 0.9% saline from VWR (Radnor, PA). Triazole 1.1 [32] and triazole 187 [39] were synthesized as previously described [32, 39] and were at >99% purity for all studies as determined by high performance liquid chromatography (HPLC). For all in vitro assays, compounds were prepared in DMSO at concentrations spanning from 32 nM to 10 mM, for dilutions with the final DMSO concentration of less than 1% in any assay.

### Cell lines and cell culture

Chinese hamster ovary (CHO, ATCC) cells expressing human kappa opioid receptor (hKOR) complementary DNA (cDNA) including three haemagglutinin tags on the N-terminus (3×HA-hKOR, cDNA.org) were generated and used as previously described; the DiscoveRx PathHunter^TM^ U2OS hKOR (U2OS-hKOR-βarrestin2-EFC) were purchased from DiscoveRx Corp. (now Millipore Sigma, Burlington, MA) and are used as previously described [22, 32–34, 39, 40].

### ^35^S-GTPγS binding assay

Cell membranes are prepared from CHO-hKOR cells and assessed for [^35^S]-GTPγS binding following treatment with KOR agonists according to previously published protocols [32, 34]. Two technical replicates were included in each individual experiment (*n* ≥ 3).

### Forskolin-stimulated cAMP accumulation assay

The CISBIO homogeneous time-resolved florescence (HTRF) cAMP kit (CISBIO HTRF cAMP Gs HiRange from Revvity) was used to assess cAMP levels as previously described in detail [34]. HTRF ratios (fluorescence at 665 nm/ fluorescence at 620 nm) were determined using a BioTek Synergy Neo2 Hybrid Multimode reader (Agilent, Santa Clara, CA) as a measure of inhibition of forskolin (20 µM) cAMP accumulation. Three technical replicates were included in each individual experiment (*n* ≥ 3).

### βArrestin2 recruitment assay

βArrestin2 recruitment is determined using the DiscoveRx PathHunter enzyme complementation assay from MilliporeSigma in PathHunter U2OS OPRK1 βarrestin-2 cells according to manufacturer’s protocol and as previously described [32, 34, 40]. Luminescence values are determined by using a BioTek Synergy Neo2 Hybrid Multimode reader. All compounds were run in triplicate per assay and normalized to vehicle-treated cells; three technical replicates were included in each individual experiment (*n* ≥ 3).

### In-cell western ERK1/2 phosphorylation

In-cell western ERK1/2 phosphorylation assays are performed according to previously published protocol [32, 40, 41]. Permeabilized cells are incubated with primary antibodies for phosphorylated ERK1/2 (1:300, Cell Signaling (Beverly, MA) #4370) and total ERK1/2 (1:400 mouse, Cell Signaling #4696) at  4°C overnight in blocking buffer. After washing, secondary antibodies (Li-Cor; anti-rabbit IRDye800CW, 1:500; anti-mouse IRDye680LT, 1:1500 from LICORbio^TM^ (Lincoln, NE)) are then added in 1:1 Li-Cor blocking buffer in PBS, containing 0.025% Tween20 for 1 h at 25 °C. Florescence is measured using an Odyssey M Imaging System from LICORbio^TM^ at 700 and 800 nm. The fold ERK1/2 phosphorylation over vehicle levels was determined by first normalizing phosphorylated ERK1/2 levels to total ERK1/2 levels within each well and then normalizing to the average of the vehicle treated cells within each experiment; three technical replicates were included in each individual experiment (n ≥ 3).

### Radioligand binding assays

Membrane preparations are made after CHO-hKOR cell homogenization and subjected to radioligand binding assays according to a modified procedure to previously published protocols [32, 42]. Membranes (20 μg protein/well) are incubated for 2 h at 25 °C in the presence of 2 nM ^3^H-Naloxone (Revvity, USA, Concentration = 1000 μCi/mL, SA = 79.9 Ci/mmol) and test compounds with a final DMSO concentration of 1%. Nonspecific binding was determined for each radioligand by competition with 1 μΜ naloxone. The affinity of ^3^H-Naloxone was determined by homologous competition: K_D_ = 3.6 nM. Two technical replicates were included in each individual experiment (n ≥ 3).

### Animals

Male C57BL/6 J mice were purchased from The Jackson Laboratory. KOR-KO mice were purchased from The Jackson Laboratory and propagated using homozygous breeding as previously described [43]. Adult mice are used between 10 and 24 weeks of age and are only used once per assay. Mice are kept on a 12-h light/dark cycle in a temperature-controlled room and are group-housed (three to five mice per cage). All behavioral tests were performed during the light cycle between 8 am–6 pm. For drug treatments mice are injected with compound or vehicle at 10 μl/g body weight. Injections are either subcutaneous (s.c.) or intraperitoneal (i.p.), indicated in figures and figure legends. Experimenters conducting injections and performing measurements are blinded to the treatment groups. The vehicle for animal studies is DMSO, Tween 80, and 0.9% sterile saline (1:1:8). To facilitate solubility, the compounds are prepared by dissolving drug powder in DMSO, adding warm (50 °C) Tween80, then 0.9% saline and immediately vortexing; solutions were made and used immediately before each study. All experiments conducted with mice are approved by the Institutional Animal Care and Use Committee at The Herbert Wertheim UF Scripps Institute for Biomedical Innovation & Technology (Jupiter, FL) and adhere to the National Institutes of Health Animal Care guidelines.

### Pharmacokinetics

To determine drug levels in plasma and brain; male C57BL6/J mice were injected with triazole 1.1 (10 mg/kg, s.c.) or triazole 187 (5 or 15 mg/kg, i.p.); plasma was taken at the indicated time points in figures. For brain levels, following cervical dislocation, brains were taken at 30, 60, 120, or 240 min and frozen in liquid nitrogen. Samples were subjected to Liquid chromatography (Shimadzu)–tandem mass spectrometry from AB Sciex (Framingham, MA) operated using multiple reaction monitoring with a mass transition of 429.3→97.1 Da for triazole 187, and 431.3→173.2 Da for triazole 1.1. Pharmacokinetic parameters were calculated using a noncompartmental model (Phoenix WinNonlin, Pharsight Inc.).

### Pruritus

The mouse pruritus assay was conducted as previously described [22, 44], using a chloroquine phosphate-induced scratching model in mice that has been validated for the assessment of pruritic behavior as previously described [45]. Mice are briefly acclimated to clear acrylic testing boxes (10 × 10 cm^2^) for 1 h then injected with vehicle, U50,488H, triazole 1.1 or triazole 187 and returned to the acrylic boxes and video recording is started. Ten minutes after pretreatment, mice receive an injection of 40 mg/kg chloroquine phosphate (CP) from Sigma-Aldrich. CP is freshly prepared in a solution consisting of DMSO, Tween80 and 0.9% saline (1:1:8); first dissolved in 0.9% sterile saline and brought up to volume with DMSO and Tween80 to; pH 6.0 and injected s.c. in the skin at the base of the neck at a volume of 5 μl/g body weight. Mice are immediately placed back into the acrylic boxes, and the number of scratching bouts is counted for 1 h by an investigator blinded to the treatment groups. During the experiments, mice are videotaped and another investigator repeated scoring to validate method. One “scratching bout” is defined as one or more rapid movements of the hind paw toward the injection site before placing the paw back on the floor [46].

### Diuresis

KOR agonists typically induce diuresis in humans and in mice [24]. To assess urinary output, mice are habituated in the experimental room for 1 h prior to the experiment with access to water, after which they are handled and weighed. For drug administration, investigators are blinded to treatments and doses, and the mice are injected s.c. with KOR agonist or vehicle and placed in 1/3 of a new (home) cage (18 × 12 × 12 cm) (separated with a divider) with the bedding replaced by hydrophobic sand (LabSand, Coastline Global, Inc., West Chester, PA). Mice are monitored for the duration of the 3 h test and urine is immediately collected from the sand upon output into a 1.5 mL tube which is kept closed to prevent evaporation. Total volume is measured at the end of the test by a calibrated pipettor. Subcutaneous administration is used in this assay for all compounds to prevent unintended pressure to the bladder.

### Climbing behavior

During the diuresis assay, investigators observed mice climbing to the top of the home cage; in subsequent diuresis assays, these attempts were recorded. An attempt is defined as every time a mouse tried to climb out of its cage during the 3 h study and the experimenter had to direct the mouse back into its cage. If a mouse reached for the top of the cage wall but did not bring its body to the top it is not considered an attempt. The doses are the same as described for diuresis.

### Locomotor activity

Dose-dependent reductions in ambulatory behavior, measured using the locomotor activity assay in mice, have been reported to correlate with drug-induced sedative-like effects observed in higher species[35–37]. Therefore, we measured changes in unhabituated activity in the open field using the Versamax Animal Activity Monitoring System (20 × 20 × 30.5 cm) from Accuscan Instruments (Columbus, OH), using Versadat software as previously described [22, 44]. Without habituation, mice are injected with vehicle, U50,488H, triazole 1.1 or triazole 187 (dose and route indicated in figures) and placed immediately into the locomotor motor activity box after injection to record spontaneous locomotor activity over 60 min. Measurements of distance traveled, number of beam breaks, time spent in the center, and center distance traveled in 5-min intervals are automatically recorded and are presented as the sum of measures over test time or in some cases, the individual time points over the 60 min period.

### Mouse nesting

Nest building is a natural behavior in mice that is sensitive to disruption by many CNS activating drugs, including KOR agonists [47–53]. To assess the effect of KOR agonists in mice, a mouse nesting assay was conducted similarly to previously described [49]. Briefly, mice are singly housed in a new (home) cage for three days for habituation before the start of the nesting study. Following habituation, mice are subject to 2 days of nesting sessions (one per day) to acclimate them to the experimental conditions and the procedure room. A nesting session entails briefly removing the mouse from the home cage and placing nestlets into 6 zones of the home cage. The mouse is then returned to the cage, and an investigator measures the number of zones cleared over 1 h. Clearing of a zone occurs when the nestlet is moved (usually to make one pile in a corner) [49]. On day 3 mice are given saline, placed in their home cage for 10 min, briefly removed to allow for placing of the nestlets in 6 zones, then returned to the home cage for nesting. Mice that failed to clear more than 3 nestlets after the saline injection were not used for further analysis. On day 4, investigators are blinded to treatments where mice are injected with vehicle, U50,488H, triazole 1.1, or triazole 187; photographs were collected and another blinded investigator also contributed to validating scoring.

### Elevated plus maze

To assess anxiolytic-like behavior in mice we utilized the elevated plus maze, an assay designed to assess anxiety related behaviors in rodents [54]. Mice are placed into a structure shaped like a “plus sign”, in which two of the arms are exposed and two are enclosed with walls (black maze with white flooring; Med Associates, St. Albans, VT) as previously described [54]. Mice are injected (i.p) with KOR agonists or vehicle and returned to their home cages for 30 min before placing in the center of the plus maze and allowed to explore the maze for 5 min. Time spent in proximal and distal areas of each arm is electronically recorded for 5 min using EthoVision XT video recording from Noldus Information Technology Inc. (Leesburg, VA). Mice are excluded if they fell off the maze or if total activity is more than two standard deviations less than mean vehicle-treated activity. The elevated plus maze was performed by the Animal Behavior Core at The Herbert Wertheim UF Scripps Institute for Biomedical Innovation & Technology and the investigator was blinded to treatment. Normalization of time spent is based on the 5 min test duration as maximum.

### Statistical and data analyses (in vitro)

All statistical comparisons are made using GraphPad Prism 10 software (GraphPad Software Inc., San Diego, CA) and is expressed as means ± SEM, unless indicated otherwise. For in vitro cell-based functional assays, agonist stimulation is determined and presented by normalizing all values to the top of the maximum response (E_max_) produced by U69,593 and using the baseline within the assay to determine the bottom. The values of half maximal effective concentration from individual experiment and (pEC_50_) and E_max_ values are derived from a three-parameter, nonlinear regression analysis on the experimental replicates and are presented as the mean with 95% CI in Table 1. A comparison of potency (logEC_50_) was performed within Prism using the extra sum-of-squares *F* test (*p* = 0.05) to compare the curves derived from of at least three independent experiments per agonist. Biased agonism calculations are described below. For radioligand binding assays, pK_*i*_ values are compared between U69,593 and each triazole using an extra sum-of-squares F test using GraphPad Prism to compare the global fit values derived from the curve of at least three independent experiments for each agonist. Individual experiments were performed in duplicate and normalized to vehicle-treated cells; data are presented as means of 3 or more individual experiments (mean with S.E.M. data are graphed; EC50 values with 95% CI are presented in the table).

#### Calculation of signaling bias

Bias of the test ligands was determined as previously described [34, 42]. This form of bias analysis employs a reference ligand that is assumed to be a full neutral agonist (i.e., the reference agonist activates all response pathways equally and does not exhibit a preference for one pathway over another). U69,593 serves as the reference agonist. ΔLogR values were determined for each experiment and these values were averaged to generate a mean ΔLogR as previously described [55]. ΔΔLogR values were calculated by subtracting the mean ΔLogR from assay 2 (βarrestin assay) from the similarly calculated mean ΔLogR from assay 1 (GTPγS binding or cAMP inhibition) and is presented as the mean with the 95% confidence intervals. All calculations were performed with GraphPad Prism 10 software; ΔΔLogR values were calculated by an unpaired *t* test to generate confidence error. Bias factors were calculated as the antilog of the ΔΔLogR [56, 57]:

### Statistical and data analyses (in vivo)

For most of the behavioral studies, vehicle and U50,448H treatments serve as controls and statistical comparison groups are indicated in the figure legends. For determination of potency, data are normalized using the vehicle response and the maximum response to U50,488H to generate the maximum possible effect (%MPE). In some cases, the maximum dose of U50,488H produced confounding sedative effects; therefore, for normalization of center time and climbing, the maximum doses of U50,488H were excluded for normalization and nonlinear regression (as indicated in Table 1). Dose response curves of the %MPE data are fit to a hyperbolic function due to the imposed maximum limitation, using GraphPad Prism, wherein the normalized maximum response produced by U50,488H (100%) is used to constrain the maximum fit of the curve. Potency calculations (ED_50_) and the comparison of the highest doses (%MPE) are presented in the table with 95% confidence intervals with statistical analysis. Comparison of potency values are determined by applying an extra sum-of-squares *F* test (*p* = 0.05) between two curves within the nonlinear regression analysis using Graphpad Prism; comparisons of %MPE in the table are made by ordinary one-way ANOVA. In the figures, raw data are presented as the individual animals and with means ± S.E.M.; effects are compared to vehicle by one-way ordinary ANOVA with a Dunnett’s post-hoc comparison; comparison between drugs is made by Tukey post-hoc comparison, significance was set at *p* < 0.05. Animal numbers are indicated in the figure legends.

## Results

### Pharmacological and pharmacokinetic characterization of Triazole 187

**Fig. 1:**
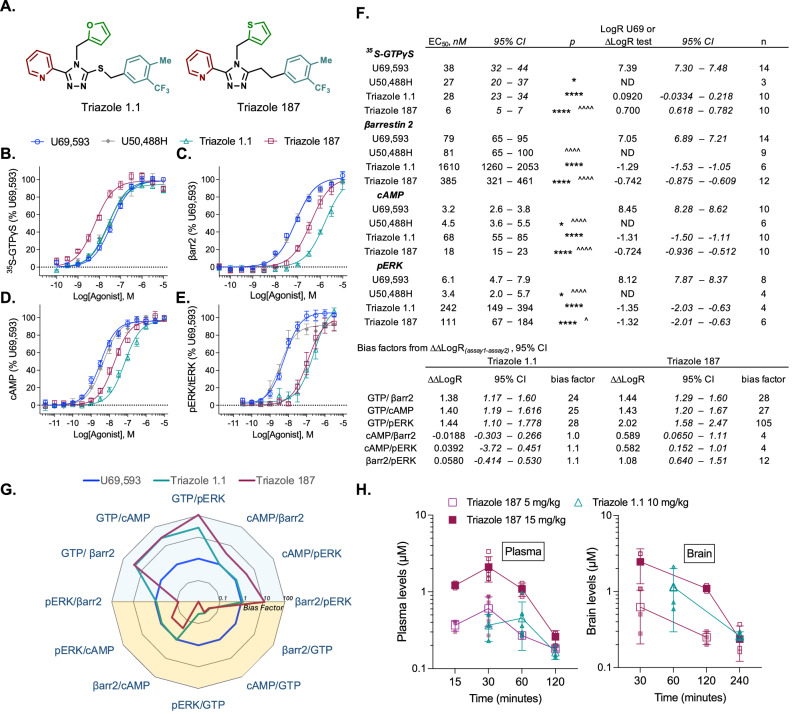
Pharmacological and pharmacokinetic characterization of triazole 187 compared to U50,488 and triazole 1.1. **A** Structures of triazole 1.1 and triazole 187. **B** ^35^S-GTPyS binding; **C** βarrestin2 recruitment; **D** inhibition of forskolin-stimulated cAMP accumulation; and **E** ERK1/2 phosphorylation are presented as % maximal U69,593 response. Data are presented as mean ± SEM; potencies with 95% CI and *n* are detailed in **F**. **F** Pharmacological parameters (mean with 95% CI) and bias factors are presented where bias factors were calculated using U69,593 as the reference agonist to define unity. ND: not determined, *p < 0.05, ****p < 0.0001; Extra sum of squares *F* test (*p* = 0.05) comparing curves of replicates to U69,593; ^*p* < 0.05; ^^^^*p* < 0.0001 Extra sum of squares *F* test (*p* = 0.05) comparing curves of replicates to triazole 1.1. **G** Bias factors are plotted on a logarithmic scale (base 10) (10^ΔΔLogR) in the web plot; as presented, a value of >10 indicates a preference for the numerator. **H** Pharmacokinetic properties of triazole 187 compared to triazole 1.1 measured in C57BL6/J male mice: shown are plasma (left) and brain (right) levels after intraperitoneal injection (i.p.) over time at the doses indicated presented as mean ± SD (*n* = 3 brains; *n* = 3 plasma except *n* = 6 triazole 1.1 s.c. at 60 min and *n* = 6 for triazole 187 i.p. at 30 min).

Cellular signaling assays were undertaken to compare triazole 187 to triazole 1.1, U50,488H and U69,593. U69,593 serves as the reference agonist for biased signaling comparisons and U50,488H. Triazole 187 shows improved affinity over triazole 1.1 and U69,593, in [^3^H]-Naloxone competition binding assays (pK_*i*_ ± S.E.M., M: U69: 9.15 ± 0.084; Tri1.1: 9.03 ± 0.084; Tri187: 9.63 ± 0.13; compared to triazole 187: *p* = 0.0002 vs Tri1.1; *p* = 0.0027 vs U69, *n* = 3) (Supplemental Fig. 1A). Triazole 187 is more potent than triazole 1.1 and U69,593 for promoting [^35^S]-GTPyS binding, inhibiting forskolin-stimulated cAMP accumulation, and recruiting βarrestin2 to KOR (Fig. 1B–D, 1F). In ERK1/2 phosphorylation, triazole 187 does not gain potency relative to triazole 1.1 but retains poor potency relative to U69,593 (Fig. 1E, F). To compare the relative potency between the assays, a bias factor analysis was undertaken, using U69,593 as the reference agonist, as previously described [22, 34, 55] (Fig. 1F,G). While the potency of triazole 187 improves, the overall bias for inducing GTPγS binding over the other signaling assays remains.

**Table 1:** ED_50_ for mouse behavior assays in Figs. 2–4.

MALE MICE:	ED_50_ mg/kg	95% CI	p vs U50	p vs 1.1
***Pruritis***				
U50,488H	0.17	*0.086–0.36*		
Triazole 1.1	0.58	*0.30–1.11*	0.0059	
Triazole 187	0.16	*0.027–0.69*		0.0341
***Diuresis***				
U50,488H	7.6	*4.8–12*		
Triazole 1.1	87	*50–188*	<0.0001	
Triazole 187	57	*32–122*	<0.0001	
***Climbing^***				
U50,488H	1.3	0.33*–*3.5		
Triazole 1.1	41	21*–*91	<0.0001	
Triazole 187	17	7.6*–*46	<0.0001	
***Nesting***				
U50,488H	0.85	*0.45–1.4*		
Triazole 1.1	25	*13–49*	<0.0001	
Triazole 187	9.2	*4.7–19*	<0.0001	0.0332
***Sedation (distance)***				
U50,488H	3.3	*2.1–5.0*		
Triazole 1.1	nc			
Triazole 187	80	*37–266*	<0.0001	
***Sedation (beam)***				
U50,488H	3.1	*2.0–4.6*		
Triazole 1.1	nc			
Triazole 187	130	*64–495*	<0.0001	
***Center Time^***				
U50,488H	1.9	*0.16–11*		
Triazole 1.1	nc			
Triazole 187	5.6	*2.6–11*	<0.0001	0.0092
***Center Distance***				
U50,488H	3.1	*1.9–4.9*		
Triazole 1.1	nc			
Triazole 187	56	*27–148*	<0.0001	

FEMALE MICE:	ED_50_ mg/kg	95% CI	p vs U50
***Sedation (distance)***			
U50,488H	5.5	*2.6–11*	
Triazole 187	29	*16–60*	0.0006
***Sedation (beam)***			
U50,488H	5.7	*3.6–8.9*	
Triazole 187	33	*19–60*	<0.0001
***Center Time^***			
U50,488H	nc		
Triazole 187	1.0	*0.15–3.1*	
***Center Distance***			
U50,488H	4.3	*1.9–9.0*	
Triazole 187	122	*43–∞*	<0.0001

ED_50_: Extra sum of squares *F* test (*p* = 0.05) comparing the normalized %MPE curves.

%MPE compared to U50,488H by unpaired t-test of the highest dose.

^a^for Climbing, the max dose used is 10 mg/kg U50,488H;

^b^for Center time, the max dose used is 15 mg/kg of U50 for normalization and %MPE comparison.

Triazole 187 was tested for off-target binding at 42 potential receptor targets by the NIMH-Psychoactive Drugs Screening Program (PDSP) which, did not reveal any high affinity targets other than KOR (Supplemental Fig. 1B) [58]. Triazole 187 (5 and 15 mg/kg i.p.) was also evaluated for brain and plasma levels in C57BL6/J mice (Fig. 1G); like triazole 1.1 (10 mg/kg, s.c., which was first published in [22, 32] and repeated here); triazole 187 is also brain penetrant and can still be detected in brain at 4 h (Fig. 1H). For comparison, striatal levels of U50,488H at 30 min post injection are 2.0 ± 0.3 µM after a 5 mg/kg, s.c. dose and 8.1 ± 1.9 µM after a 15 mg/kg, s.c. dose (mean with SD, *n* = 3) [22].

### Triazole 187, triazole 1.1, and U50,488H are potent anti-pruritic compounds

**Fig. 2:**
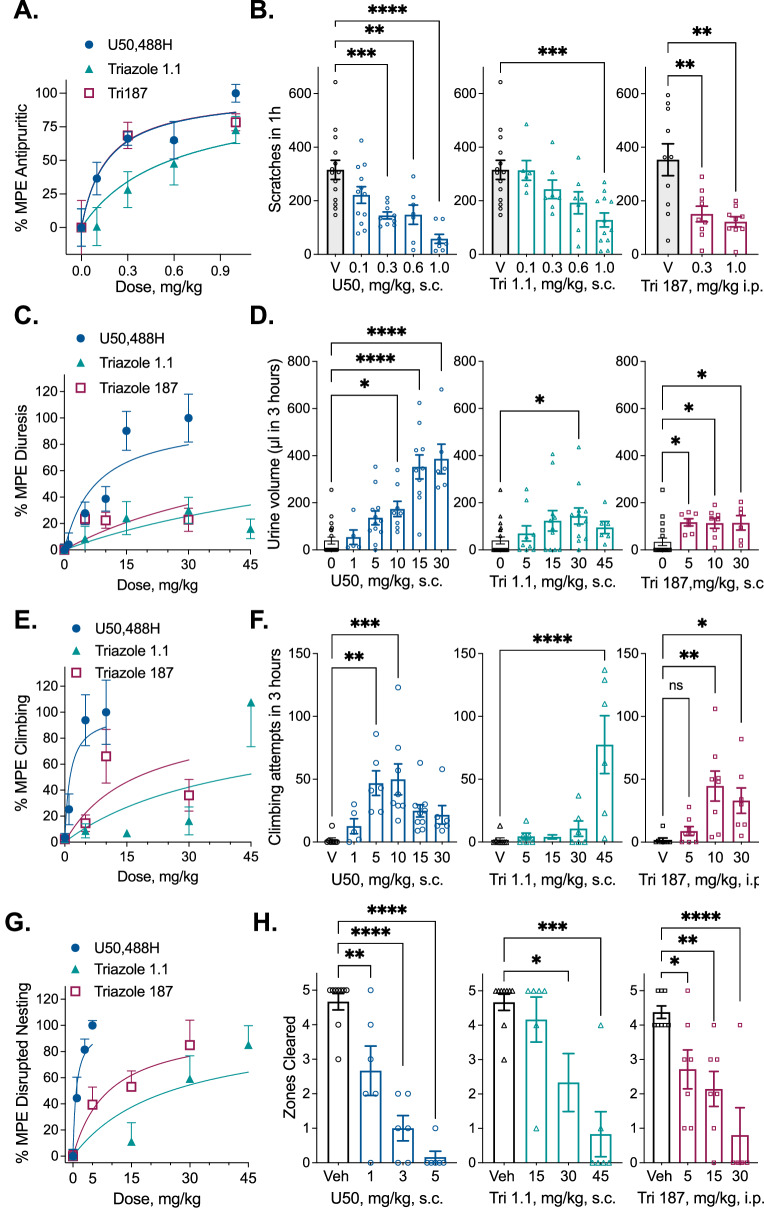
KOR agonists effects on chloroquine phosphate-induced pruritus diuresis, climbing behavior and nesting behavior in male C57BL6/J mice. **A** U50,488H, triazole 1.1, and triazole 187 dose-dependently suppress chloroquine-phosphate (CP) (40 mg/kg, s.c. neck) induced scratching in in male C57BLJ/6 mice when administered 10 min before CP. **B** The number of scratches are shown (*n*: Veh s.c.: 14; U50 s.c.: 7-12; Tri 1.1 s.c.: 6-12; Veh i.p.: 10 Tri 187 i.p.: 9). **C** U50,488H, induces more urine production than triazole 1.1 and triazole 187 over a 3 h period compared to vehicle. **D** The urinary output in µL is shown per dose (*n*: Veh s.c.: 21; U50: 5-12; Tri 1.1: 6-12; Tri 187: 6-8). **E** Climbing responses were counted during the urine collection over 3 h. Since U50,488H is sedative at higher doses, the 10 mg/kg dose was used to set the maximum and 15 and 30 mg/kg data were not fit to determine potency in the %MPE curve. **F** Climbing attempts are shown for each dose (*n* are: the same as in **D**). **G** U50,488H potently disrupts nesting behaviors. **H** The number of zones cleared are shown for each dose (*n*: Veh s.c.:9; Veh i.p.: 8; U50: 6; Tri 1.1: 6-7; Tri 187: 5-7). The calculated ED_50_ from the hyperbolic curves for each compound is presented in Table 1 with 95% CI. Data are presented as mean ± S.E.M. Dose vs. vehicle comparisons were conducted using ordinary one-way ANOVA with Dunnett’s post-hoc test (*p < 0.05, **p < 0.01, ***p < 0.001, ****p < *0.0001*).

Triazole 1.1 was previously shown to be as equally efficacious as U50,488H in the chloroquine phosphate-induced pruritus mouse model when compared at a dose of 1 mg/kg [22]. A dose response study, utilizing lower doses of the compounds, was undertaken to determine the median efficacious dose (ED_50_) (Fig. 2A,B, Table 1). The ED_50_ for triazole 187 is not different from U50,488H and represents an improvement over triazole 1.1 (see Table 1 for statistical comparisons of ED_50_ values). U50,488H and triazole 187 suppresses scratching at 0.3 mg/kg, while only the highest (1.0 mg/kg) dose of triazole 1.1 produces a significant effect relative to vehicle treatment (Fig. 2B).

### Triazole 1.1 and triazole 187 produce less diuresis in mice

Diuresis is a common side effect of typical KOR agonists [24]. In mice, U50,488H, triazole 1.1 and triazole 187 increase urine output over a 3-h period (Fig. 2C, D); however, triazole 1.1 and triazole 187 appear to be less efficacious at higher doses than U50,488H (Fig. 2C, Table 1). Notably, the total urine volume produced by the highest doses of triazole 1.1 (95 ± 26 µL) and triazole 187 (115 ± 31 µL) is significantly less compared to that induced by U50,488H (386 ± 63 µL) (vs U50: tri 1.1, *p* = 0.0007; tri 187, *p* = 0.0015, one-way ordinary ANOVA *p* = 0.0004, *n* = 6 per group; Fig. 2D). While a mild diuretic effect remains, the overall impact is less pronounced for the two biased agonists compared to U50,488H, even as the dose is increased.

### U50,488H and triazole 187 induce climbing behavior in mice

While monitoring mice for diuresis, we observed mice attempting to climb out of the assessment box (12 cm wall height); these attempts were recorded over the 3-h test period. The ED_50_ of U50,488H was less than either triazole for increasing climbing behaviors (Fig. 2E, F, Table 1). Since higher doses of U50,488H are known to be sedative [22, 23, 59], the effects at 15 and 30 mg/kg were not included in determining the ED_50_ of U50,488H (Fig. 2E, Table 1).

### Triazole 1.1 and triazole 187 produce less disruption of nesting behaviors than U50,488H

Nest building is an instinctive behavior in mice that can be disrupted by drugs that induce both pleasant and aversive effects in humans, including cannabis, alcohol, morphine and antidepressants [47–52]. KOR agonists have also been shown to disrupt nesting behavior [49, 53]. Doses of U50,488H were chosen to avoid sedative-like effects based on previous studies [22]. As anticipated, U50,488H potently disrupts mouse nesting behavior at doses as low as 1 mg/kg (Fig. 2G, H, Table 1). While the triazoles interfere with nesting behaviors at higher doses, they are significantly less potent (higher ED_50_) than U50,488H (Fig. 2G, Table 1).

### Triazole 1.1 and triazole 187 do not induce pronounced sedation in mice

**Fig. 3:**
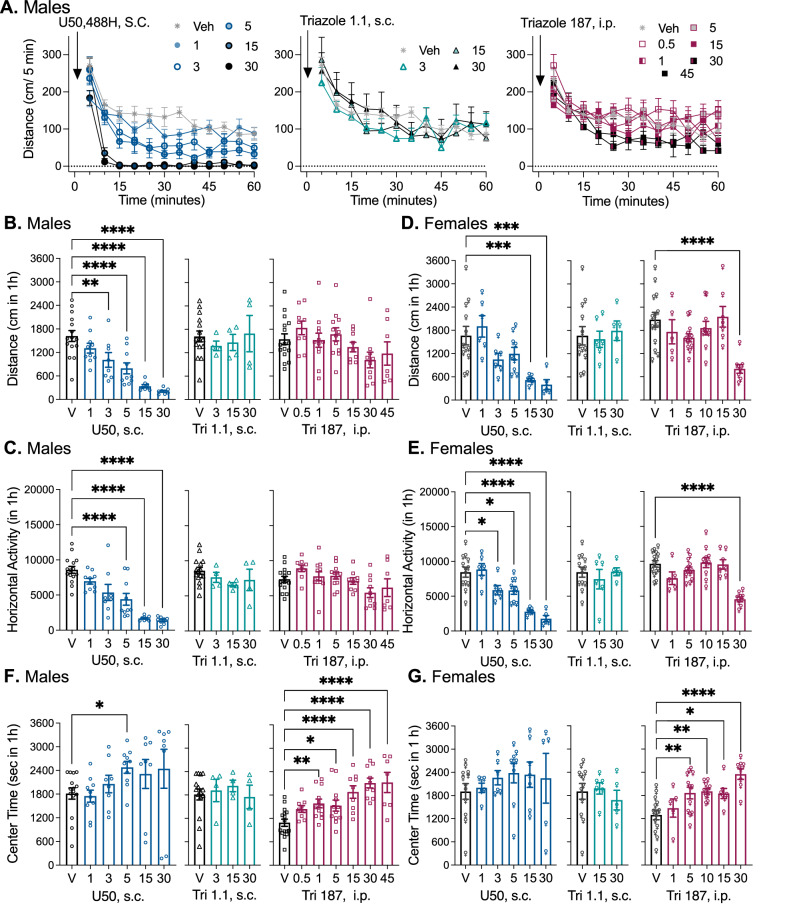
Triazole 187 increases center time but does not induce sedation in male and female C57BL6/J mice. **A** The distance traveled in male C57BL6/J is rapidly decreased by U50,488H (s.c.) while triazole 1.1 (s.c.) and triazole 187 (i.p.) do not produce this effect over time (time x dose, 2-way RM-ANOVA U50,488H: F_(29.15, 309)_ = 2.501, *p* < 0.0001; triazole 1.1: F_(15.87, 121.6)_ = 0_._9740, *p* = 0.4888; triazole 187: F_(66, 748)_ = 1.279, *p* = 0.0734). **B** The sum of the total distance traveled over 1 h and (**C**) the sum of horizontal activity (beam breaks) from the same mice are compared. **D** Female mice are compared for total distance and (**E**) horizontal beam breaks. **F** Comparison of time spent in the center compartment for male and (**G**) female mice. (males: *n* = Veh s.c.: 15; Veh i.p.: 16; U50: 8-10; Tri 1.1: 4; and Tri 187: 7-12; females: *n* = Veh s.c.,12; Veh i.p., 14; U50: 6-9; Tri 1.1: 6; Tri 187:10-12). Dose vs. vehicle comparisons were conducted using ordinary one-way ANOVA with Dunnett’s post-hoc test (*p < 0.05, **p < 0.01, ***p < 0.001, ****p < *0.0001*). Triazole 1.1 and U50,488H were administered s.c. and their vehicle is given via the same route. Triazole 187 and its vehicle were administered by the i.p. route. Potency (ED_50_) values with 95% CI and 2-way RM-ANOVA analyses for time are presented in Table 1.

In mice, sedative drugs decrease spontaneous locomotor activity in an open field [50, 60]. We extended our analysis to determine the median efficacy dose (ED_50_) and also to consider other measures captured by the infrared activity monitors. Consistent with our prior studies, U50,488H rapidly decreases the distance traveled over 1 h in a dose responsive manner (Fig. 3A, time x dose, 2-way RM-ANOVA U50,488H: F_(29.15, 309)_ = 2.501, *p* < 0.0001) and significantly decreases total distance traveled compared to vehicle at doses higher than 3 mg/kg (one-way ANOVA, Fig. 3B). This is recapitulated in horizontal activity as measured by the number of beam breaks occurring in the open field (Fig. 3C). Triazole 1.1 and triazole 187 (Fig. 3A-C) do not produce this effect over time (time x dose, 2-way RM-ANOVA triazole 1.1: F_(15.87, 121.6)_ = 0.9740, *p* = 0.4888; triazole 187: F_(66, 748)_ = 1.279, *p* = 0.0734) and do not significantly decrease the distance traveled or horizontal activity at any dose tested relative to vehicle in male mice (Table 1). The sedating effects of U50,488H are selective for KOR as KOR-KO mice do display this effect [22] (Supplemental Fig. 2).

The compounds were also tested in female mice, as KOR agonists have been reported to be less potent in females compared to male mice in models that assess effects on mood modified behaviors [61–64]. In female mice, U50,488H decreases the distance traveled at 15 and 30 mg/kg, s.c. (Fig. 3D), although decreases in the horizontal activity (Fig. 3E) mirrors the effects of U50,488H observed in the males (Fig. 3C). Comparable to the males, total distance and horizontal activity in female mice are not decreased by triazole 1.1. While having no effect at doses up to 15 mg/kg, triazole 187 produces some decrease in activity when given at 30 mg/kg in female mice and this is seen in distance traveled and horizontal activity (Fig. 3D, E, Table 1).

### Triazole 187 and U50,488H increase time spent in the center of the open field

The open field activity measures were also analyzed for time spent in the center of the field, as an indicator of anxiolytic-like behavior in mice, based on the animal´s innate aversion to novel open areas [65]. In males, U50,488H increases center time at 5 mg/kg (Fig. 3F, Table 1); while movement within the center zone is significantly decreased at 3, 5, 15 and 30 mg/kg (Supplemental Fig. 3A, Table 1), mirroring the total distance traveled and horizontal activity (Fig. 3A, B). Triazole 1.1 does not affect the time spent in the center compared to its vehicle at any dose tested (Fig. 3F) nor does it affect the distance traveled within the center zone (Supplmental Fig. 3A). Triazole 187 increases center time at several doses compared to its vehicle (Fig. 3F) without decreasing overall distanced traversed in the center (Supplemental Fig. 3A, Table 1). The effects of triazole 187 and U50,488H on center time and center distance are absent in KOR-KO mice (Supplemental Fig. 2).

In female mice, U50,488H-induced increases in center time does not reach statistical significance at any of the doses tested; however, center distance traveled is decreased by U50,488H at 15 and 30 mg/kg (Fig. 3G, Supplemental Fig. 3B, Table 1). Recapitulating the results in the males; triazole 1.1 produces no changes in center time or center distance in female mice. In contrast to U50,488H, triazole 187 significantly increases center time in females at 5, 10 and 15 mg/kg, although at 30 mg/kg, a decrease in center distance is observed (Fig. 3G, Supplemental Fig. 3B, Table 1).

### Triazole 187 increases time spent in open arms of the elevated plus maze

**Fig. 4:**
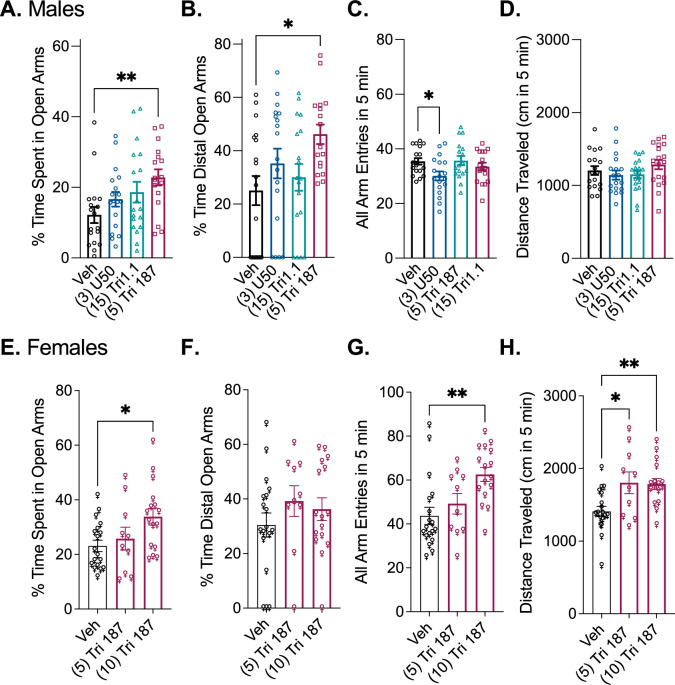
Triazole 187 increases time spent in open arms of the elevated plus maze in male mice and increases all arm entries and distance traveled in female mice. Vehicle (i.p.), U50,488H (3 mg/kg, i.p.), triazole 1.1 (15 mg/kg, i.p.) or triazole 187 (5 mg/kg, i.p.) were administered to male mice (**A**–**D**) and vehicle or triazole 187 (5 mg/kg or 10 mg/kg, i.p.) were given to (**E**–**H**) female mice 30 min prior to the start of the 5-min test. Measures are presented as the percent of time spent in the open arms; the percent of time spent in the distal part of the open arm; the total number of arm entries; and the total distance traveled in the entire maze in 5 min. Data are presented as mean ± S.E.M. **A** Males: *n* = Veh i.p.: 18; U50: 18; Tri 1.1: 18; and Tri 187: 17; drug vs vehicle: (*p < 0.05, **p < 0.01 by one-way ordinary ANOVA, with Dunnett’s post-hoc test). **B** females: *n* = Veh i.p.: 18; and Tri 187: 10-16; triazole 187 compared to vehicle: (*p < 0.05, **p < 0.01 by one-way ordinary ANOVA, with Dunnett’s post-hoc test).

An elevated plus maze was used to further investigate anxiolytic-like behaviors induced by the different agonists in male (Fig. 4A–D) and female (Fig. 4E–H) mice. Mice were administered doses based on the locomotor activity assay results. A dose of 3 mg/kg U50,488H, was chosen because it increases center time without decreasing horizontal activity, and 5 mg/kg triazole 187, a dose that is approximately the ED_50_ for the center time effect (Table 1). A higher dose of Triazole 1.1 (15 mg/kg) was chosen in hopes of reaching efficacy without concern for sedation (Fig. 3). In males, the 3 mg/kg dose of U50,488H decreases the total number of arm entries but it does not decrease the total distance traversed; moreover, it does not increase time spent in the open arms. Not unexpectedly, triazole 1.1 has no effect on any of the parameters measured. Triazole 187 (5 mg/kg), however, increases the time spent in the open arms and in the distal end of the open arms without affecting arm entries or overall activity (Fig. 4A–D). In female mice, 5 mg/kg of triazole 187 increased overall exploratory activity (distance traveled). At 10 mg/kg, triazole 187 led to a significant increase in the time spent in the open arms, the frequency of enter the arms and increased the total distance traversed compared to vehicle (Fig. 4E–H). While higher doses were needed to see an increase in the open arm time in females, it appears that triazole 187 retains anxiolytic-like properties in both male and female mice.

## Discussion

Triazole 187 is a member of a new series of compounds [39] that shows improved affinity, potency and preserved selectivity and bias for promoting GTPγS binding to KOR over several other KOR mediated signaling pathways. In mice, triazole 187 is more potent in suppressing chloroquine phosphate-induced itch than triazole 1.1, while retaining a lack of sedation, suggesting that potency can be improved while preserving the widened therapeutic window. While diuresis is induced by all the agonists tested, the overall volume produced by triazole 1.1 and triazole 187 is less than half as much as U50,488H, suggesting that this adverse effect may also be diminished for these agonists. Unlike U50,488H, triazole 187 increases time spent in the center region of the open field while not producing sedation, potentially indicating anxiolytic-like effects of the biased KOR agonist.

Clinically, KOR agonists are prescribed for the treatment of non-histamine pruritus that can accompany hemodialysis [3]. However, non-dermatitis, non-inflammatory itch is difficult to treat; moreover, its condition has been described as tormenting to endure, leading to severe impacts on mood. Chronic itch often produces anticipation and anxiety in patients about future symptoms, which can contribute to the worsening of the condition [66, 67]. Extensive studies show that psychiatric comorbidities, including anxiety and depression, are commonly found in patients with chronic pruritus. These patients also experience high rates of sleep disturbances and report a significantly lower quality of life [68–72]. Our results present a promising strategy, by CNS-permeable biased KOR agonists, for advancing the treatment of pruritus and the psychological comorbidities that can accompany this condition. Aside from pruritus, our data suggests that biased KOR agonists may be useful for the treatment of anxiety in general, the anxiolytic studies conducted herein were performed in naïve mice (i.e., those not experiencing intractable itch).

It is known that activation of KOR in the CNS produces psychoactive effects [10, 73, 74] and modulates various mood states, such as anxiety and depression [10, 37, 75–78]. KOR antagonists have been pursued for treating neuropsychiatric conditions, with recent advancements in drug development aimed at addressing stress-related mood disorders and major depressive disorders [79–81]. In contrast, conventional KOR agonists are generally not considered viable clinical interventions due to the adverse effects associated with their use. However, a biased KOR agonist may present a new means to modulate mood without sedation.

A number of behavioral observations were included in this study as a general assessment of the impact of the biased agonist on general behaviors. In the open field, unlike U50,488H, triazole 187 does not lead to dose-dependent decrease in spontaneous locomotor activity in male mice. While reductions in ambulatory activity are associated with sedative-like drug effects in higher species [35–37], such decreases can also arise from changes in exploration, anxiety, pain, motivation, or motor coordination [82, 83]. We also observed an increase in climbing behavior induced by U50,488H, as well as triazole 187. Enhanced climbing behavior has previously been observed with U69,593 in mice [84] and could indicate novelty seeking and exploring [83]. On the other hand, climbing behavior may be representative of drug-induced escape behavior [85]. In this study, we also found that U50,488H disrupts mouse nesting behavior at a sub-sedative dose (1 mg/kg), while triazole 187 and triazole 1.1 are significantly less potent than U50,488H (Table 1). Nesting behavior can be disrupted by both positive and negative stimuli [47–52], making it challenging to fully interpret these results as favorable or aversive [86]. Regardless, there remains a significant reduction in potency for the biased agonists relative to U50,488H in the nesting and the climbing studies (Table 1).

Prior studies have shown that the conventional KOR agonists, such as U50,488H, produce anxiolytic-like effects in rodents in the elevated plus maze [12], but since assessments of anxiolytic-like behaviors in mice measure movement, the sedative effects induced by these compounds can confound interpretation [87, 88]. In this study, upon using lower doses of U50,488H (1 and 3 mg/kg) to avoid sedation, we were unable to observe increases in center time in the locomotor activity assay and increases in time spent in the open arms of the elevated plus maze. Conversely, triazole 187, which does not induce sedation, increases in center time and demonstrates efficacy in the elevated plus maze in both male and female mice, potentially indicating anxiolytic-like effects. Notably, these drug effects can be observed in the same cohort of mice after drug treatment, as both the open-field activity and elevated plus maze simultaneously measure the overall distance traveled and time spent in the center of the open-field or the open arms of the maze. However, additional tests, including light/dark box and marble burying test will be required to further validate these anxiolytic-like effects in rodents. Taken together, our study demonstrates that the diverging behavioral effects of a biased agonist can be differentiated within the same cohort of animals in a dose dependent manner; we show that anxiolytic behaviors can be enhanced without sedation.

KOR agonists have produced different effects between the sexes in rodent models of mood modulation. For example, decreased responses to the dysphoria-like effects of U50,488H, were noted in female rats in intracranial self-stimulation (ICSS) studies [64, 89] and female mice were less responsive to U50,488H in anxiety-related behaviors and conditioned place aversion (CPA) [53, 90]. Additionally, female mice require a higher dose of U50,488H to suppress nesting, a preclinically measure of overall well-being, further suggesting that females are less sensitive to KOR mediation compared to males [53]. We find that while U50,488H dose-dependently decreases horizontal activity in the open-field in female mice, we did not observe a significant increase in center time. We also noted that triazole 187 retains efficacy in both sexes in for increasing center time and also for spending time in the open arms; although a higher dose (10 mg/kg) was needed in the females compared to males. Interestingly, the lower 5 mg/kg dose produced more overall activity in the elevated plus maze which could be a manifestation of overall exploratory behaviors, although open arm time was not increased at this dose in the females. Taken together, triazole 187 shows favorable anxiolytic-like effects in both male and female mice without the added confound of sedation.

Aside from sedation, another disruptive side effect of KOR agonists is diuresis, which occurs in both mice and humans [24, 91–93]. Moreover, the clinically used difilikefalin, despite its peripheral restriction, still induced significant diuresis which is mediated by actions in the CNS [94]. Additionally, nalfurafine has been reported to induce diuresis in rats [95], while U50,488H has been reported to induce diuresis in rats, as well as mice and non-human primates [24, 59, 92, 96]. Triazole 1.1 and 187 induce a significant increase in diuresis, however, the output is significantly less (~30%) than that induced by U50,488H. This mild diuretic effect appears to approach a ceiling effect and could be seen as clinical advantage in developing a mild diuretic or as analgesics for pain accompanied by edema.

Overall, this study highlights and contributes to the growing literature demonstrating that it is possible to separate therapeutic benefits from side effects by imparting selectivity for different receptor active states. However, while these findings strengthen the evidence that avoiding KOR:βarrestin2 interactions may lead to KOR agonists with fewer side-effects, the results remain correlative, as it is not clear whether βarrestin2 is actually driving the onset of adverse effects. Moreover, the triazole agonists also display bias against signaling via other secondary effectors, such as adenylyl cyclase and ERK1/2 (Fig. 1) [34, 62], and these separations may prove to be important. Finally, our studies are restricted to correlative behaviors in mice and may vary in other species, including humans. With the gain of anxiolytic properties, triazole 187 represents a new generation of G protein signaling-biased KOR agonists that may improve the future development of non-sedating antipruritic agents and anxiolytics. Additionally, CNS-permeable biased KOR agonists, such as triazole 187, present a promising alternative therapeutic approach for advancing the treatment of chronic itch as well as the anxiety often associated with this condition.

## Supplementary information

supplemental figures

Volf et al., 2026 NPP data from Figures

## Data Availability

The individual data points used in analysis are shown in the bar chart figures; when mean data are presented, that the raw data can be found in the extended data set.
