# Supplementary material for: Triazole 187 is a biased KOR agonist that suppresses itch without sedation and induces anxiolytic-like behaviors in mice
Source: Neuropsychopharmacology. 2026 Mar 9;51(8):1485–96. doi: 10.1038/s41386-026-02372-8 (PMC13291347; doi:10.1038/s41386-026-02372-8)
Supplement: Supplementary file 1 — supplemental figures [file 41386_2026_2372_MOESM1_ESM.pdf]

Supplemental Material for:

Triazole 187 is a biased KOR agonist that suppresses itch without sedation and induces anxiolytic-like behaviors in mice.

Allison Volf[1,2] , Tarsis F. Brust[2], Robin R. Kobylski[1,2], Kerri M. Czekner[2,3], Edward L. Stahl[3], Michael D. Cameron[1], Ashley E. Trojniak[4], Abbey B. Wood[4], Jeffrey Aubé[4*], and Laura M. Bohn[1,3*].

1 - The Skaggs Graduate School at Scripps Research, La Jolla, CA and Jupiter, FL

2 - Department of Molecular Medicine, The Herbert Wertheim UF Scripps Institute for Biomedical Innovation & Technology - Jupiter, FL 33458 – USA

3- Department of Molecular Pharmacology and Physiology, Morsani College of Medicine, University of South Florida

4 - Division of Chemical Biology and Medicinal Chemistry, UNC Eshelman School of Pharmacy, University of North Carolina at Chapel Hill - Chapel Hill, NC 27599 – USA

*Correspondence should be addressed:

JAube@email.UNC.edu or LBohn@usf.edu

A.

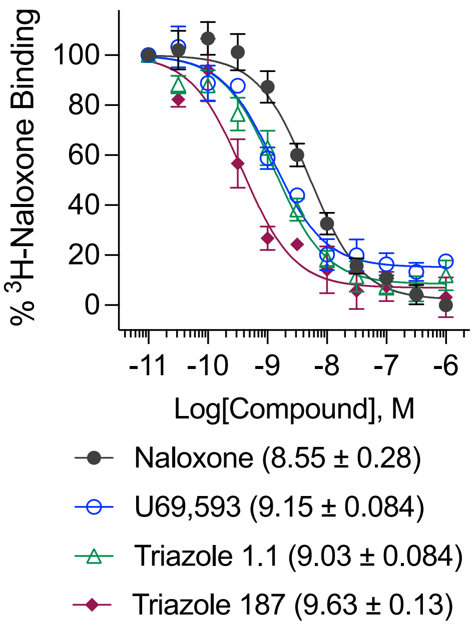

B.

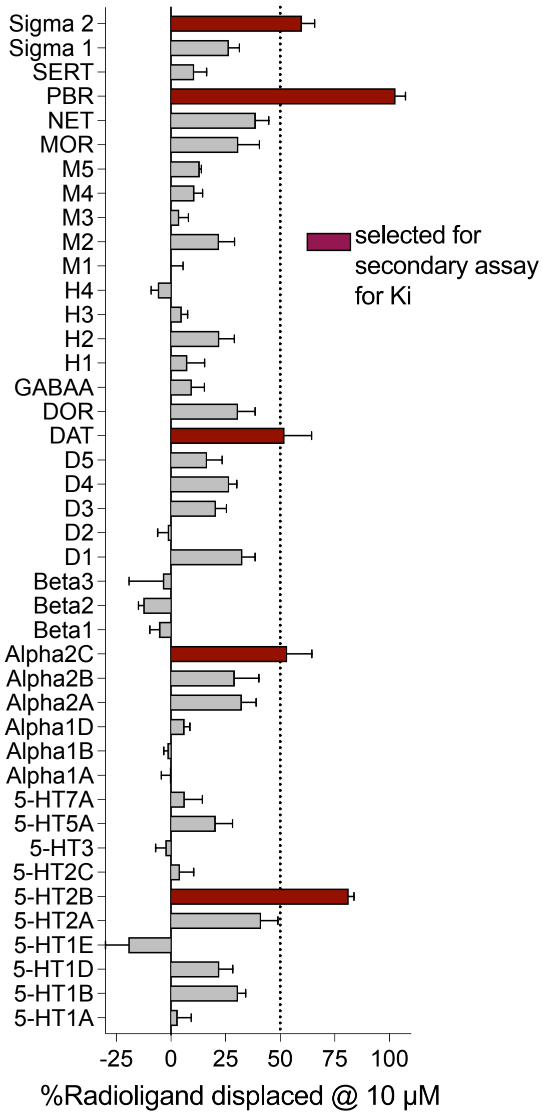

C.

Secondary Competition binding for Ki

**Receptor**	**Tri 187 Ki (nM)**	**Radioligand**	***Radioligand Kd (nM)***
Alpha2C	3811.54	[³H]-Rauwolscine	*0.96*
5-HT2B	883.69	[³H]-LSD	*1.19*
DAT	582.37	[³H]-Win35428	*9.4*
PBR (rat brain)	386.1	[³H]-PK11195	*1.0*
KOR	5.81	[³H]-U69593	*0.83*
Sigma 2	1437.81	[³H]-Ditolylguanidine	*9.9*

Figure S1. KOR affinity and selectivity in psychoactive drug target screening of Triazole 187. (A) Binding affinity comparing U69,593, Triazole 1.1, Triazole 187 and naloxone by competition binding using 2 nM ^{3}H-Naloxone as the radioligand (the pK_D for naloxone and the pK_i with S.E.M. are presented in the figure). **(B)** The Psychoactive Drugs Screening Program (PDSP) tested the ability of Triazole 187 to displace radioligand binding of 42 psychoactive drug target proteins at a 10 μM concentration. Five receptor targets showed displacement greater than 50% and were further investigated for competition binding assays to determine affinity. **(C)** Binding affinity determined by competition binding. The K_D of the radioligand used in the competition assays is provided for comparison (from the PDSP).

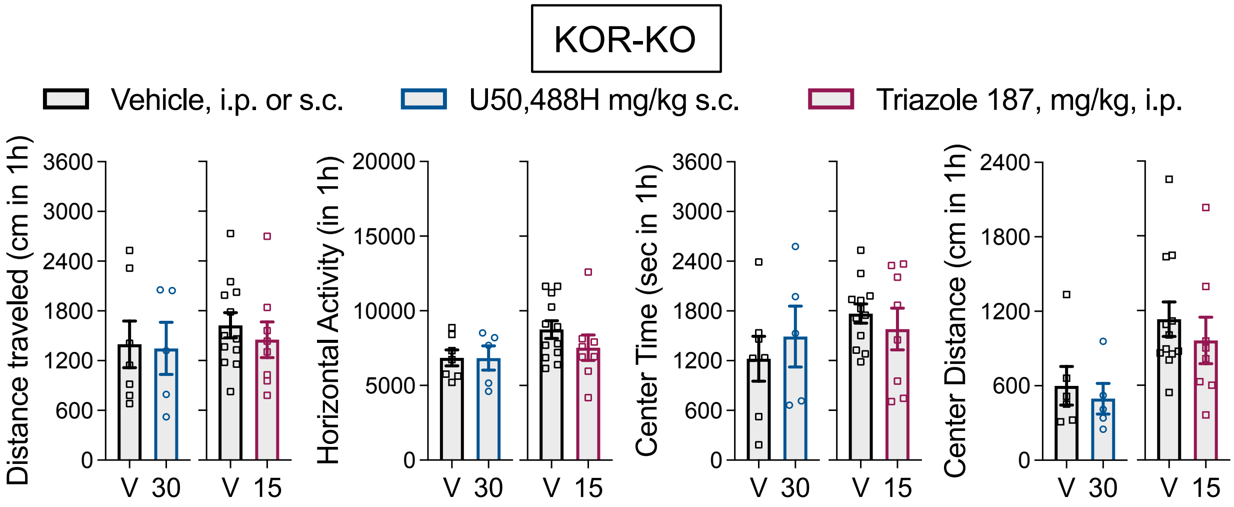

Figure S2. KOR agonists effects on spontaneous locomotor activity in KOR-KO. In male KOR-KO mice distance travelled, horizontal activity, center time and center distance did not differ between vehicle and drug treated (unpaired t test, $p>0.05$). (KOR-KO: male *n* = Veh s.c., 7; Veh i.p., 12; U50, 5; Tri 1.1, 7; Mean and s.e.m. are plotted with individual animals shown by symbols.

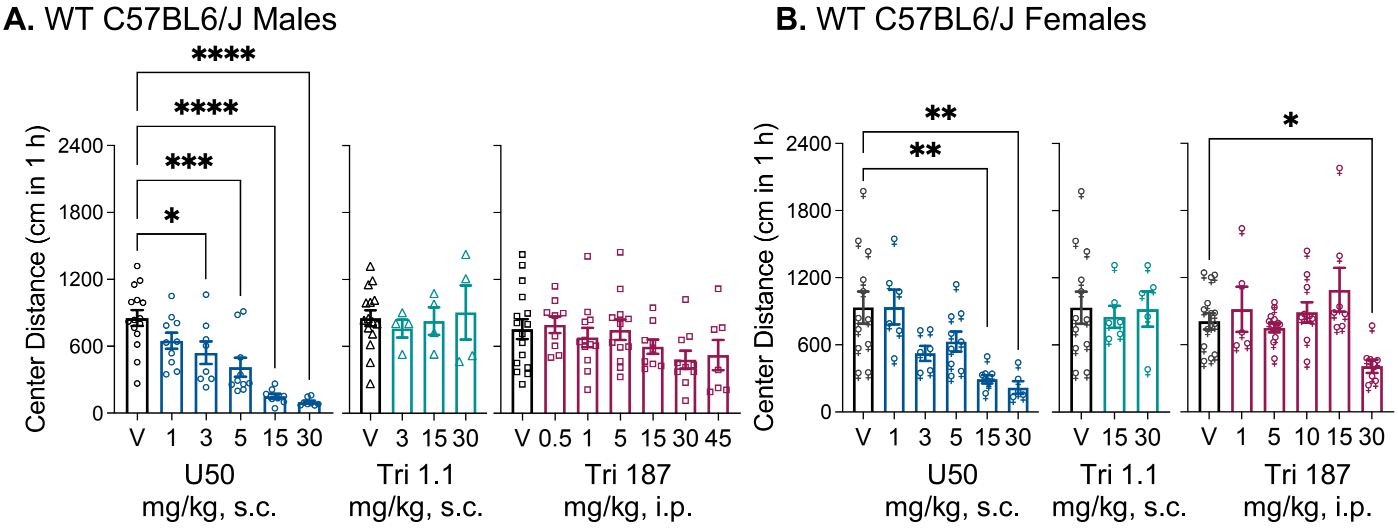

Figure S3. U50,488H decreases distance travelled in the center in male and female C57BL6/J mice. (A) U50,488H decreased total distance travelled in the center of the open-field over 1-hour compared to vehicle**;** triazole 1.1 and triazole 187 had not effect on center distance travelled. **(B)** In female C57BL6/J mice U50,488H decreased total distance travelled in the center of the open-field over 1-hour at 15 mg/kg U50,488H compared to vehicle**,** triazole 1.1 and triazole 187 had not effect on center distance travelled. Data are presented as mean ± SEM and potencies are presented with 95% CI in Table 2. (male C57BL6/J: *n* = Veh s.c., 15; Veh i.p., 16; U50, 8-10; Tri 1.1, 4; and Tri 187, 7-12; female C57BL6/J: *n* = Veh s.c.,12; Veh i.p., 14; U50, 5-9; Tri 1.1, 5-6; and Tri 187, 6-12). Drug vs. vehicle comparisons were conducted using ordinary one-way ANOVA with Dunnett's post-hoc test (**p<0.05, **p<0.01, ***p<0.001, ****p<0.0001*). Triazole 1.1 and U50,488H were administered s.c. and their vehicle is given via the same route. Triazole 187 and its vehicle were administered by the i.p. route. Potency (ED_{50}) values with 95% CI for center distance are presented in Table 1.
